# LRP8 is overexpressed in estrogen‐negative breast cancers and a potential target for these tumors

**DOI:** 10.1002/cam4.1923

**Published:** 2018-12-21

**Authors:** Virginie Maire, Faisal Mahmood, Guillem Rigaill, Mengliang Ye, Amélie Brisson, Fariba Némati, David Gentien, Gordon C. Tucker, Sergio Roman‐Roman, Thierry Dubois

**Affiliations:** ^1^ Translational Research Department, Institut Curie PSL Research University Paris France; ^2^ Breast Cancer Biology Group Paris France; ^3^ Institute of Plant Sciences Paris‐Saclay (IPS2), UMR 9213, UMR1403 CNRS, INRA, Université Paris‐Sud, Université d’Evry, Université Paris‐Diderot, Sorbonne Paris‐Cité Orsay France; ^4^ Laboratoire de Mathématiques et Modélisation d’Evry (LaMME) Université d’Evry Val d’Essonne, UMR CNRS 8071, ENSIIE, USC INRA Evry France; ^5^ Preclinical Investigation Laboratory Paris France; ^6^ Genomics Platform Paris France; ^7^ Center for Therapeutic Innovation in Oncology, Institut de Recherches SERVIER Croissy‐sur‐Seine France

**Keywords:** apoptosis, HER2, LRP8, targeted therapy, triple‐negative breast cancer

## Abstract

Triple‐negative breast cancer (TNBC) is the breast cancer subtype with the worst prognosis. New treatments improving the survival of TNBC patients are, therefore, urgently required. We performed a transcriptome microarray analysis to identify new treatment targets for TNBC. We found that low‐density lipoprotein receptor‐related protein 8 (LRP8) was more strongly expressed in estrogen receptor‐negative breast tumors, including TNBCs and those overexpressing HER2, than in luminal breast tumors and normal breast tissues. LRP8 depletion decreased cell proliferation more efficiently in estrogen receptor‐negative breast cancer cell lines: TNBC and HER2 overexpressing cell lines. We next focused on TNBC cells for which targeted therapies are not available. LRP8 depletion induced an arrest of the cell cycle progression in G1 phase and programmed cell death. We also found that LRP8 is required for anchorage‐independent growth in vitro, and that its depletion in vivo slowed tumor growth in a xenograft model. Our findings suggest that new approaches targeting LRP8 may constitute promising treatments for hormone‐negative breast cancers, those overexpressing HER2 and TNBCs.

## 1 INTRODUCTION

Breast cancer is a heterogeneous disease, and its various subtypes can be considered as different diseases, with different clinical outcomes. Transcriptomic analysis has identified four main breast cancer subtypes: those expressing the estrogen (ER) and/or progesterone (PR) receptors (luminal A and luminal B tumors, the latter being more proliferative), and those not expressing them (basal‐like and ER^−^/HER2^+^ tumors, the latter overexpressing human epidermal growth factor receptor 2, HER2).^1^, ^2^ Basal‐like tumors are a highly heterogeneous group^3^, ^4^, ^5^, ^6^, ^7^ resembling triple‐negative breast cancers (TNBCs), which are characterized by immunohistochemistry (IHC) by their lack of ER/PR expression and of HER2 overexpression.^8^, ^9^ The therapeutic management of breast cancer has greatly improved in recent years, but prognosis remains poor for TNBC patients.^8^, ^9^ These tumors respond well to current therapeutic strategies based on conventional chemotherapy, but they continue to account for a large proportion of breast cancer deaths, because of their high rate of recurrence from residual, resistant tumor cells.^9^ There are currently no targeted treatments for TNBC, and this disease remains a major challenge for oncologists. Alternative treatments are therefore needed, to bypass chemoresistance and to improve the survival of TNBC patients.^4^, ^5^, ^8^, ^9^, ^10^, ^11^, ^12^

Low‐density lipoprotein (LDL) receptor‐related protein 8 (LRP8), also known as apolipoprotein E receptor‐2 (APOER2), belongs to a superfamily of single‐pass transmembrane receptors comprising LDL receptors (LDLR) and LDLR‐related proteins (LRPs).^13^, ^14^ LRP8 displays high levels of sequence identity (50% in for the amino acid sequence) with the very‐low‐density lipoprotein receptor (VLDLR).^13^ These two receptors have Reelin as a common ligand.^15^ LRP8 regulates neuronal differentiation and migration^13^, ^14^ and also functions in the brain, as a receptor for the cholesterol transport protein apolipoprotein E (ApoE), which has been reported to be a genetic risk factor of Alzheimer's disease.^16^ LRP8 has also been reported to be a novel activator of the Wnt/β‐catenin signaling pathway during osteoblast differentiation.^17^ Very few reports to date link LRP8 to cancer initiation and/or progression.^18^, ^19^, ^20^, ^21^

We found that LRP8 was more strongly expressed (at the mRNA level) in breast cancers without hormone receptor expression (TNBC [ER^−^/PR^−^/HER2^−^], ER^−^/HER2^+^) than in luminal tumors (luminal A [ER^+^/HER2^−^], luminal B [ER^+^/HER2^+^]), and normal breast tissues. Using two different siRNAs, we showed that LRP8 depletion impaired more strongly the proliferation of estrogen receptor‐negative (TNBC and ER^−^/HER2^+^) breast cancer cell lines compared to the luminal cell lines. LRP8 depletion promoted apoptosis and impaired cell proliferation in the tested cell lines. Furthermore, LRP8 knockdown impaired colony formation in an anchorage‐independent assay (soft agar), suggesting that LRP8 has tumorigenic properties. These findings were further confirmed by experiments showing that LRP8 depletion slowed tumor growth in an in vivo xenograft model. Overall, our results identify LRP8 as a new treatment target in hormone‐negative breast cancers, including ER^−^/HER2^+ ^and TNBCs, for which new treatments are urgently required due to the high risk of relapse after chemotherapy.

## 2 MATERIALS AND METHODS

### 2.1 Human samples and microarray data

Our cohort, comprising 35 luminal A (LA: ER^+^/HER2^−^), 40 luminal B (LB: ER^+^/ HER2^+^), 46 TNBC (ER^−^/PR^−^/ HER2^+^), 33 ER^−^/PR^−^/HER2^+^, and 18 normal breast tissues, has been described elsewhere.^22^, ^23^ The RNA microarray (Affymetrix U133 plus 2.0, Paris, France) results have also been reported elsewhere.^22^, ^23^, ^24^, ^25^

The TCGA breast invasive carcinoma (TCGA‐BRCA) cohort is publicly available.^26^ The RNA‐SeqV2 Level 3 data (Jan 2015) were downloaded from the TCGA Research Network (http://cancergenome.nih.gov/) and integrated into a platform in knowledge data integration (KDI) at Institut Curie (https://bioinfo-portal.curie.fr). Subtype classification was based on immunohistochemical status for the estrogen receptor (ER), progesterone receptor (PR), and HER2, as follows. TNBC: ER‐, PR‐, and HER2‐negative (n = 157); HER2^+^/ER^−^: ER‐ and PR‐negative, HER2‐positive (n = 41); luminal B: ER‐ and/or PR‐positive, HER2‐positive (n = 153); and luminal A: ER‐ and/or PR‐positive, HER2‐negative (n = 663).

The Molecular Taxonomy of Breast Cancer International Consortium (METABRIC) dataset (Illumina data)^27^ was used as another validation set. Data were downloaded and analyzed as described.^28^ Subtype classification was as follow: TNBC: ER‐, PR‐, and HER2‐negative (n = 319); HER2^+^/ER^−^: ER‐ and PR‐negative, HER2‐positive (n = 133); luminal B: ER‐ and/or PR‐positive, HER2‐positive (n = 115); and luminal A: ER‐ and/or PR‐positive, HER2‐negative (n = 1422).

### 2.2 Cell lines, cell authentication, and cell culture

Cell lines were purchased from the American Type Culture Collection (ATCC, LGC Promochem, Molsheim, France).

All cell lines used in this study were authenticated and validated (data not shown) in 2018 using the PowerPlex® 16 System (Promega, Charbonnieres les bains, France). Briefly, genomic DNA was extracted from cell pellet using a Nucleospin kit (Macherey‐Nagel, Hœrdt, France). An RNAse A step was added to the DNA extraction process. Genomic DNAs were controlled by a direct quantification method using a NanoDrop device (ND8000; Thermo, Courtaboeuf, France) to evaluate their purity and using an indirect method (Qubit; Thermo) to determine the concentration of double‐stranded DNA. Then, 1 ng of genomic DNA was used to amplify the 16 short tandem repeats included in the Powerplex 16 system. Amplicons were detected and analyzed using an ABI PRISM® 3500XL Genetic Analyzer (Applied Biosystems, Villebon‐sur‐Yvette, France; 24‐capillaries, 50 cm length, matrix: pop7). The STR analysis matching was carried out using the DSMZ dedicated web page (http://www.dsmz.de/fp/cgi-bin/str.html).

Cells were cultured as previously described.^22^, ^23^, ^24^, ^25^, ^29^ BT‐474, T‐47D, MDA‐MB‐468, and ZR75‐1 cells were maintained in RPMI‐1640 (Life Technologies) supplemented with 10% (vol/vol) fetal bovine serum (FBS; Life Technologies, Courtaboeuf, France), 100 U/mL penicillin, and 100 µg/mL streptomycin (P/S; Life Technologies). The same media complemented with 1.5 g/L sodium bicarbonate (Life Technologies), 10 mmol/L HEPES (Life Technologies), and 1 mmol/L sodium pyruvate (Life Technologies) was used for HCC1143, HCC1569, HCC1954, HCC38, and HCC70 cells. MDA‐MB‐453 cells were cultured in DMEM‐F12 (Life Technologies) supplemented with 10% FBS and P/S. BT‐20 and MCF7 cells were cultured in MEM (Sigma‐Aldrich, Saint‐Quentin Fallavier, France) containing 10% FBS, P/S, 1.5 g/L sodium bicarbonate, 0.1 mmol/L nonessential amino acids (NEAA; Life Technologies), and 1 mmol/L sodium pyruvate. SK‐BR‐3 cells were cultured in McCoy5a (Life Technologies) containing 10% FBS and P/S. We maintained all cell lines at 37°C in a humidified atmosphere with 5% CO_2_.

### 2.3 Small interfering RNAs (siRNAs) and transfection

Cells were transfected with siRNAs as previously described.^22^, ^23^, ^25^, ^29^ Cells were seeded into 6‐ or 96‐well plates at a density determined on the growth rate of each cell line (Table S1). Forward transfection was performed for T‐47D, BT‐20, MCF‐7, MDA‐MB‐468, MDA‐MB‐453, SK‐BR‐3, HCC1143, HCC1569, and HCC1954 cells with 20 nmol/L siRNA duplexes, using INTERFERin reagent (Polyplus, Ozyme, Montigny Le Bretonneux, France) in Opti‐MEM medium (Life Technologies), according to the manufacturers’ instructions. Reverse transfection was performed at the same time of seeding for HCC70, HCC38, BT‐474, and ZR‐75‐1 cells using Lipofectamine RNAiMAX reagent (Life Technologies) in Opti‐MEM medium, according to the manufacturers’ instructions. The siRNAs (Qiagen, Les Ulis, France) used were as follows: Allstars negative control (ref SI03650318); LRP8#2 (SI00066276), target sequence: 5′‐TTGCGGAAAGGTAACCACAAA‐3′; LRP8#3 (SI00066283), target sequence: 5′‐CTGGACTGACTCGGGCAATAA‐3′.

### 2.4 Cell proliferation assays

Cell proliferation was assessed in MTT or WST‐1 assays or by real‐time monitoring with the xCELLigence Real‐Time Cell Analysis system.

The number of viable cells was determined by using a colorimetric assay based on the reduction of 3‐(4,5‐dimethylthiazol‐2‐yl)‐2,5 diphenyltetrazolium bromide (MTT; Sigma) to formazan within the mitochondria of living cells. On the day of assay, 15 µL of MTT (5 mg/mL dissolved in PBS) was added onto each well (96‐well plate) containing cells and medium. After 4‐hour incubation at 37°C, 100 µL of 10% SDS (in 10 mmol/L HCl) was added in each well and incubated at 37°C overnight. The absorbance was measured at 540 nm on an Infinite 200 spectrophotometer (Tecan, Lyon, France). For HCC38 and HCC70 cell lines, WST‐1 (Roche, Meylan, France) was added to the cells according to manufacturer's instructions. After 1‐to‐4 hour incubation at 37°C, the absorbance was measured at 440 nm on an Infinite 200 spectrophotometer (Tecan). All the experiments were performed in quadruplicate. Results are presented as percent cell viability relative to cells treated with control siRNA (100%).

Cells were seeded onto E‐plate 96 plates specifically dedicated for the xCELLigence Real‐Time Cell Analysis system (ACEA Biosciences, Ozyme, San Diego, CA). Software allowed real‐time monitoring of proliferation, morphology, size, and attachment quality of cells by measuring the impedance of electron flow which is reported using a unit‐less parameter called cell index (CI). We performed all experiments in sextuplicate, and data (*Y*‐axis) represent a relative CI that has been normalized at the time of the transfection (CI = 1).

### 2.5 Cell cycle analysis by flow cytometry

Adherent and floating cells were collected after trypsinization. The cells were then washed once with PBS followed by PBS containing 0.5% BSA, before being fixed in cold ethanol (70%). The cells were subsequently incubated in PBS containing 10 µg/mL propidium iodide (PI; Invitrogen Villebon‐sur‐Yvette, France) and 200 µg/mL RNase A (Invitrogen) for 30 minute at room temperature (RT). The data were acquired with an LSRII flow cytometer (Becton Dickinson, Le Pont de Claix, France) equipped with DIVA™ software (a minimum of 20 000 cells per sample were analyzed). The DNA content was quantified by using ModFit LT software (Verity Software House, Topsham, ME), and the results were expressed as a distribution of cells in each cell cycle phase.

### 2.6 Apoptosis evaluation

Following siRNA‐mediated depletion, the cells were harvested at different time points and the apoptosis was evaluated using the following assays:

Immunoblotting using whole protein lysates from floating and adherent cells to assay for the cleavage of PARP, caspase 7, and caspase 8, markers of cells undergoing apoptosis.
Quantification of the sub‐G1 population by using the same protocol for the analysis of cell cycle progression. The percentage of sub‐G1 population with low PI staining was determined using FlowJo software (LLC).
The percentage of apoptotic cells was also performed by using an annexin‐V‐FLUOS staining kit (Roche) following the manufacturer's instructions. After sequential staining by annexin V and PI, flow cytometry analyses were performed on a LSRII Instrument. Using FACSDiva software, minimum of 10 000 cells per sample were analyzed and the percentage of apoptotic cells with annexin‐V staining was quantified using FlowJo software.

### 2.7 Clonogenic assay

One day after siRNA transfection, the cells were trypsinized and seeded onto 6‐well plates (Table S1). The MDA‐MB‐468 cells were incubated for 10 days and HCC38/HCC70 cells for 15 days at 37°C in a humidified atmosphere with 5% CO_2. _The colonies were fixed and stained with 0.05% Coomassie Brilliant Blue R‐250, 50% methanol, and 10% acetic acid during 20 minute. The number of colonies was quantified by using the LAS‐3000 Luminescent Image analyzer (Fuji, FSVT) and ImageJ 1.43u software (NIH). All experiments were performed in triplicate. Colony counts are expressed as a percentage relative to the number of colonies obtained with cells treated with control siRNA.

### 2.8 Soft‐agar tumorigenic assay

MDA‐MB‐468 and HCC70 cells were trypsinized 24 hour following transfection with siRNA, and then resuspended (Table S1) in 0.35% soft‐agar medium, consisting of equal volumes of 0.70% agarose (Sigma) and 2× culture medium, and plated onto 1 mL of solidified 0.5% soft‐agar in 6‐well plates. MDA‐MB‐468 and HCC70 cells were incubated for 4 and 5 weeks at 37°C in a humidified atmosphere with 5% CO2, respectively. The colonies were stained with MTT and visualized. Plates were photographed with a Fujifilm LAS-3000 Imager (Fuji, FSVT), and clones were counted with Image J 1.43u software (NIH). All the experiments were performed in triplicate. Colony counts are expressed as a percentage relative to the number of colonies obtained with cells treated with control siRNA. HCC38 cells did not form colonies under these conditions (data not shown).

### 2.9 Protein extracts

The cells were lysed in Laemmli buffer containing 50 mmol/L Tris pH 6.8, 2% sodium dodecyl sulfate (SDS), 5% glycerol, 2 mmol/L 1,4‐dithio‐dl‐threitol (DTT), 2.5 mmol/L ethylenediaminetetraacetic acid (EDTA), 2.5 mmol/L ethylene glycol tetraacetic acid (EGTA), 2 mmol/L sodium orthovanadate, and 10 mmol/L sodium fluoride (Sigma‐Aldrich), a cocktail of protease (Roche) and phosphatase (Thermo Scientific) inhibitors, and then boiled at 100°C for 10 minute. The protein concentration in each sample was determined with the reducing agent‐compatible version of the BCA Protein Assay kit (Thermo Scientific).

### 2.10 Immunoblot

Equal amounts of total protein (15 or 20 µg) were fractionated by SDS‐PAGE under reducing conditions (4%‐12% TGX gels, Bio‐Rad, Marnes la Coquette, France) and blotted onto nitrocellulose membranes (Bio‐Rad). The membranes were blocked with 5% BSA in TBS containing 0.1% Tween 20 (TBS‐T) and hybridized with the primary antibody of interest overnight at 4°C. Membranes were washed in TBS‐T and then hybridized with the secondary antibody for 1 hour at room temperature. Antibodies were diluted in TBS‐T containing 5% BSA. The membranes were washed with TBS‐T, and immune complexes were revealed by enhanced chemiluminescence (SuperSignal West Pico Chemiluminescent Substrate, Thermo Scientific) and imaged using the LAS‐3000 Luminescent Image analyzer and Image Gauge software (Fuji, FSVT). The antibodies used for Western blotting were directed against beta‐actin (Sigma‐Aldrich, #A2668), cleaved caspase 7 and 8 (Cell Signaling Technology, #9491 and “9496, respectively), LRP8 and cleaved PARP (Abcam, #ab108208 and #ab108208, respectively).

### 2.11 RT‐qPCR

mRNA quantification by quantitative PCR in cell culture was performed with the QuantiTect SYBR Green RT‐PCR Kit (Qiagen), according to the manufacturer's instructions on the 7900HT Fast Real‐Time PCR System (Applied Biosystems). PCR was done from 50 ng of total RNA with QuantiTect Primer Assays targeting LRP8 and GAPDH (QT00068516 and QT00079247, Qiagen). Although we examined the expression of GAPDH, we finally did not normalize the data with this housekeeping gene, due to the fact that its expression varied a lot between the different tissues examined (Figure S1).

### 2.12 Mouse and tumor growth measurement

Five‐ to six‐week‐old female Swiss *nude* mice were purchased from Charles River laboratories (Les Arbresles, France) and maintained in specific pathogen‐free conditions. Experimental procedures were specifically approved by the Ethics Committee of the University Paris V CEEA #34 (agreement number given by National Authority: 01240.03) in compliance with the international guidelines in particular.^30^ The protocol was validated by the local ethics committee. MDA‐MB‐468 cells (4 × 10^6^) were injected subcutaneously into the mice 24 hours after transfection with control or LRP8 siRNAs (7 mice/group). Tumor growth was evaluated by caliper measurements, twice weekly, as previously described.^23^ The number of animals was minimized by investigating the effects of only one LRP8 siRNA. LRP8#3 was chosen for the analysis as it gave the highest levels of caspase activity.

### 2.13 Statistical analyses

Differences in RNA levels between groups were assessed with Student's *t* tests and considered significant if the *P* value was below 0.05. For the cell cycle experiment, we evaluated the difference between the control siRNA and the LRP8 siRNA for each cell population (G1 vs not G1, S vs not S, and G2/M vs not G2/M), in a Fisher exact test. For the in vivo experiment, we evaluated the difference between the control siRNA and the LRP8 siRNA at each time point, in a Wilcoxon test. We adjusted for multiple testing by the Benjamini‐Hochberg method for the Fisher exact and the Wilcoxon tests. Differences were considered significant if the adjusted *P* value was below 0.05.

## 3 RESULTS

### 3.1 LRP8 is highly expressed in hormone‐negative breast tumors: ER^−^/HER2^+^ and TNBC

**Figure 1:**
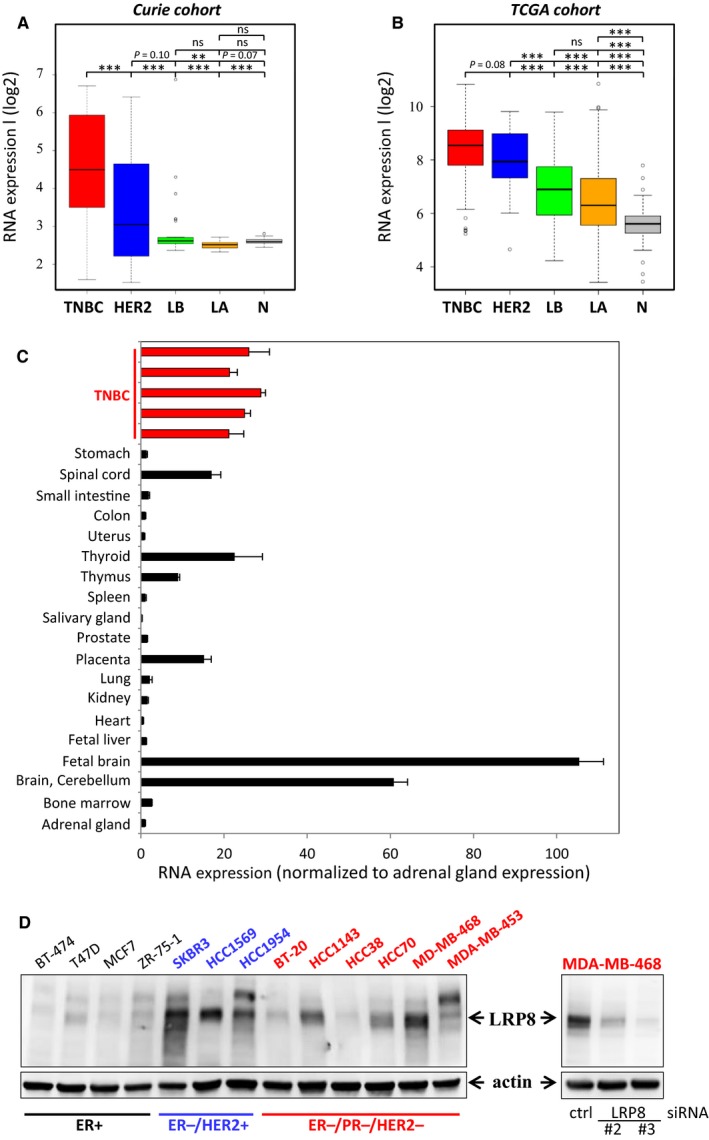
LRP8 is more strongly expressed in hormone receptor‐negative breast tumors than in luminal cancers, and expression levels are highest in TNBC. A‐B: LRP8 RNA levels in the different breast cancer subtypes. LRP8 RNA levels in (A) our cohort (Curie)^22^, ^23^ and (B) the publicly available TCGA cohort^26^; TNBC (red), HER2^+^/ER^−^ (HER2, blue), luminal A (LA, orange), and luminal B (LB, green) cancers and normal breast tissues (N, gray). The relative levels of RNA have been subjected to a logarithmic (log2) transformation and are illustrated by boxplots. Outliers are shown within each population studied (open circles). Student's *t* test was used to compare RNA levels between two groups. The *P* values are indicated (*P < 0.05; **P < 0.01; ***P < 0.001; ns *P* > 0.1). C, LRP8 RNA levels in a panel of normal tissues (black bars). Comparisons with the levels found in our cohort were facilitated by including five TNBC samples with high levels of LRP8 expression (red bars) that were investigated in (A) in this analysis. LRP8 RNA levels were normalized relative to those in the adrenal gland (=1). D, LRP8 protein levels in a panel of 13 breast cancer cell lines (left panel) and in MDA‐MB‐468 cells transfected with one of the two siRNAs against LRP8 (#2 and #3) or with a control siRNA (right panel, also presented in Figure 2A). LRP8 and actin (used as a loading control) protein levels were assessed by Western blotting and indicated by arrows. On the left panel, an upper band can be seen but is not always observed depending of the protein lysate preparation. The cells lines indicated in black express the estrogen receptor (ER^+^), those in blue do not express ER but overexpress HER2 (ER^−^/HER2^+^), and the cell lines in red do not express ER and PR, and do not overexpress HER2 (ER^−^/PR^−^/HER2^−^). Of note, MDA‐MB‐453 (red) belongs to the luminal androgen receptor (LAR) TNBC subtype

With the aim of identifying new treatment targets for TNBC, we previously generated omics data for a cohort of human samples corresponding to the various breast cancer subtypes and normal breast tissues.^22^, ^23^ We^24^, ^31^ and others^32^, ^33^ are exploring the Wnt/β‐catenin signaling pathway as a potential pathway to target for the treatment of TNBC patients. Searching for transmembrane receptors regulating the Wnt pathway that are expressed at higher levels in TNBC compared to the other breast cancers subtypes and normal breast tissues, we identified among others, LRP5,^31^ LRP6,^31^ and LRP8 (Figure 1A). LRP8 was also more strongly expressed in ER^−^/HER2^+^ tumors than in luminal tumors and normal tissues (Figure 1A). The stronger expression of LRP8 in hormone‐negative breast cancer samples (TNBC and ER^−^/HER2^+^) than in luminal tumors was confirmed in the publicly available TCGA^34^ (Figure 1B) and METABRIC (Figure S2) cohorts.

We then assessed LRP8 expression in various types of normal human tissue. Comparisons with our samples (Figure 1A) were facilitated by including some TNBCs (those with the highest levels of LRP8 expression) from our cohort into this analysis (Figure 1C). LRP8 was strongly expressed in the spinal cord, thyroid, thymus, and placenta, and its levels were highest in the brain, but most normal tissues had only low levels of LRP8 (Figure 1C). The low levels of LRP8 RNA in most normal tissues were validated by the data on the NCBI website, which reported strong expression for this receptor only in the brain, testis, and thyroid (https://www.ncbi.nlm.nih.gov/gene/7804).

In conclusion, we found that LRP8 was more strongly expressed in hormone‐negative breast cancer samples compared to luminal samples, and hypothesized that LRP8 may be required for the survival of TNBC and ER^−^/HER2^+^ cells.

### 3.2 LRP8 is required for cell survival, colony formation, and tumorigenicity

We then analyzed the effects of LRP8 depletion on cell viability. We first evaluated LRP8 protein levels in a panel of 13 breast cancer cell lines. LRP8 protein was more abundant in ER‐negative breast cancer cell lines (ER^−^/HER2^+^ and TNBC) than in ER‐positive cell lines (Figure 1D). Some variability of LRP8 protein levels was observed between TNBC cell lines, similar to that observed for LRP8 RNA levels in TNBC biopsy specimens (Figure 1A,B).

**Figure 2:**
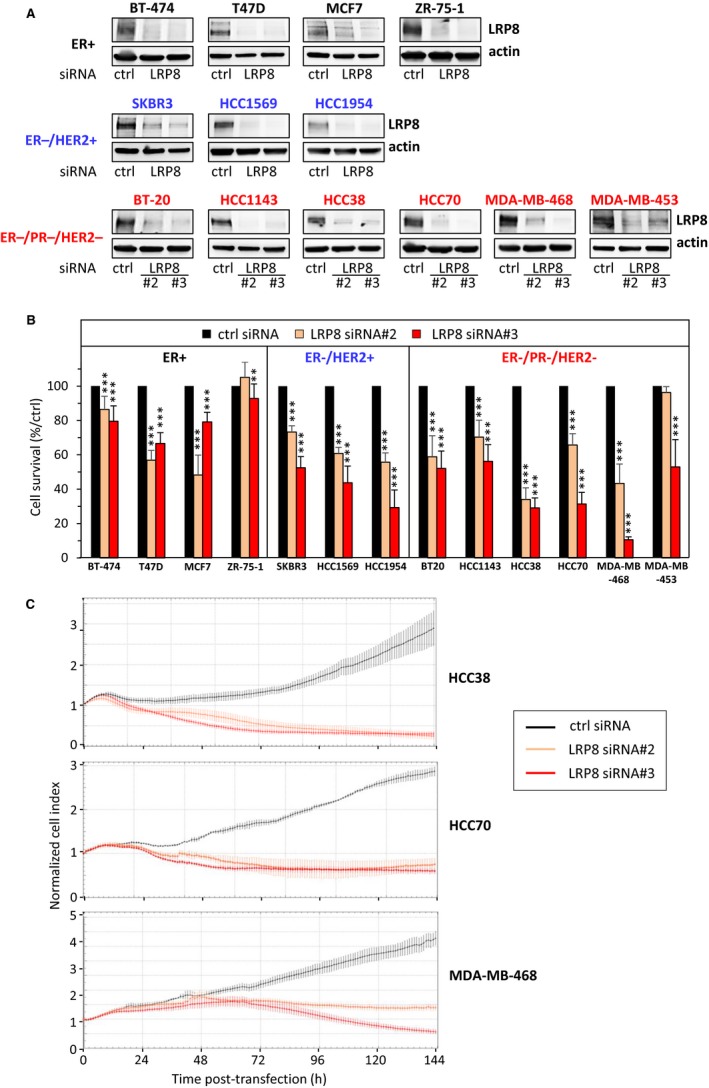
LRP8 is required for the survival of breast cancer cells. Breast cancer cell lines were transfected with one of the two siRNAs against LRP8 (#2 and #3: orange and red, respectively) or with a control siRNA (black). A, LRP8 protein levels were evaluated by Western blotting 144 h after transfection. Actin was used as a loading control. One experiment of at least two (three for MDA‐MB‐468, HCC70, HCC38, BT‐20, and MCF7), all giving similar results, is shown. B, Cell viability was assessed in MTT or WST‐1 assays, 144 h after transfection. Results are presented as percent cell viability relative to cells treated with control siRNA (100%). Data are the means + SD of at least three independent experiments. C, Dynamic monitoring of TNBC cell adhesion and proliferation with the xCELLigence system. Cell proliferation in response to siRNA treatment was monitored for 144 h (*x*‐axis), by evaluating cell index (*y*‐axis) values with xCELLigence System. The cell index was normalized relative to its value at the time of the transfection (cell index = 1 at *t* = 0)

We then investigated whether the targeting of LRP8 affected cell viability. We knocked down LRP8 expression with two different siRNAs against LRP8 (#2 and #3) in the panel of 13 breast cancer cell lines. The efficiency of LRP8 depletion was verified by Western blotting (Figure 2A). LRP8 siRNA#3 was more efficient than LRP8 siRNA #2, and this difference was particularly marked in some cell lines (SKBR3, HCC70, and MDA‐MB‐468) (Figure 2A). LRP8 depletion with both siRNAs impaired cell proliferation in most of the cell lines tested (Figure 2B). The strongest effects on cell viability were observed with LRP8 siRNA#3 (Figure 2B), the siRNA yielding the greatest depletion of LRP8 (Figure 2A). LRP8 depletion did not affect the viability of ZR‐75‐1 (ER^+^) cells and only slightly affected that of BT‐474 (ER^+^) cells (Figure 2A).

**Figure 3:**
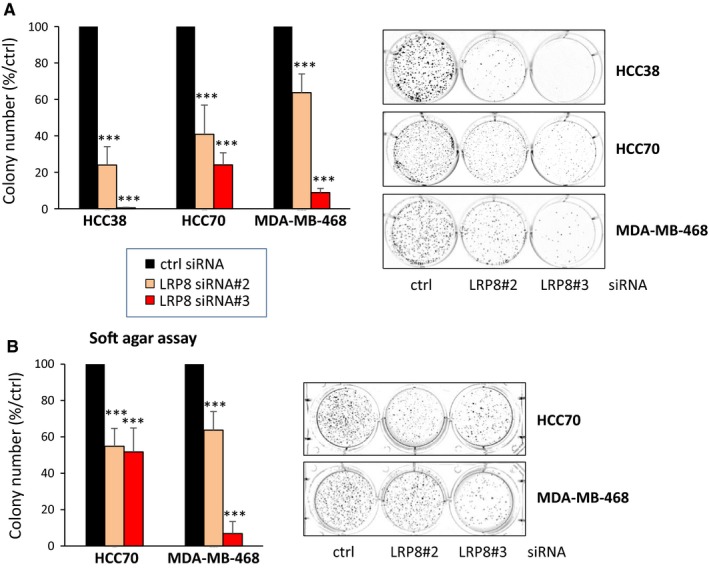
LRP8 is required for colony formation and has tumorigenic properties. TNBC cells were transfected with one of the two siRNAs against LRP8 (#2 and #3: orange and red, respectively) or with a control siRNA (black). A, Colony formation assay on plastic. Cells were transfected and transferred to six‐well plates, in which they were cultured for 10‐15 d, until colonies formed. The number of colonies is expressed as a percentage relative to that for cells treated with control siRNA (graphs). The data shown are means ± SD from three independent experiments. A representative image of one well is also shown for all conditions. B, Transfected HCC70 and MDA‐MB‐468 cells (HCC38 cells do not form colonies in this assay) were embedded in agar medium. One month later, the colonies formed were stained with MTT, photographed, and counted. Colony counts are expressed as a percentage relative to the number of colonies obtained with cells treated with control siRNA (graph). Data are expressed as means ± SD, for triplicate measurements from three independent experiments. A representative image of one well is shown for all conditions. The *P* values were determined in Student's *t* test (comparison with control siRNA): ***P < 0.001

Although LRP8 is more expressed in ER^−^/HER2^+^ and TNBC tumors and that its depletion impairs the viability of ER^−^/HER2^+^ and TNBC cells, we next focused our study on TNBC for which targeted therapies are not available. We performed additional in vitro studies on three TNBC cell lines highly sensitive to LRP8 depletion, with high (MDA‐MB‐468), medium (HCC70), and low (HCC38) levels of LRP8. Cell viability was monitored in real time after LRP8 depletion, with xCELLigence technology, and the results obtained confirmed that LRP8 was indeed required for cell survival (Figure 2C). LRP8 depletion impaired the ability of the cells to form colonies in an anchorage‐dependent (colony formation on plastic; Figure 3A) and in an anchorage‐independent (colony formation in soft agar; Figure 3B) assays. The soft‐agar assay, a well‐established method to evaluate cellular anchorage‐independent growth for the detection of the tumorigenic potential of malignant cells, was not performed with HCC38 cells, as these cells do not form colonies in these conditions. Again, the strongest effects were observed with LRP8 siRNA#3 in all cell lines.

Overall, these results demonstrate that LRP8 is essential for breast cancer cell survival and that its depletion impairs tumorigenic properties of the tested cells.

### 3.3 LRP8 controls cell cycle progression

**Figure 4:**
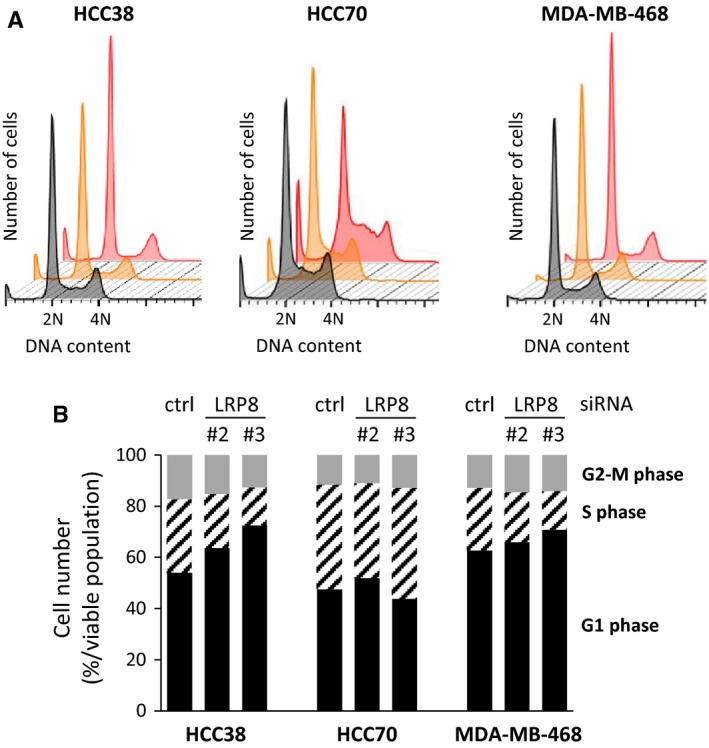
LRP8 controls cell cycle progression. A, TNBC cells were transfected with one of the two siRNAs against LRP8 (#2 and #3: orange and red, respectively) or with a control siRNA (black). We monitored cell cycle status 48 h post‐transfection, by FACS analysis of PI staining. Representative results for three independent experiments are shown. B, The percentages of cells in G1 phase (black), S phase (black hatched), and G2+ M phase (gray) are shown. The data shown are means from four independent experiments. The differences in cell populations between the control siRNA and LRP8 siRNAs were evaluated using the Fisher exact test. We used Benjamini‐Hochberg correction to adjust for multiple testing. Differences were considered significant if the adjusted *P* value was below 0.05. This was the case for all the comparisons

We then explored the molecular mechanisms by which LRP8 controls cell viability. We investigated the effects of LRP8 depletion on cell cycle progression. FACS analyses were performed in LRP8‐depleted MDA‐MB‐468, HCC38, and HCC70 cells, 48 hours after transfection (Figure 4). In LRP8‐depleted cells, the G1 population was larger and the S‐phase population was smaller than in cells transfected with the control siRNAs (Figure 4), except with HCC70 cells transfected with one of the two LRP8 siRNA (LRP8#3 siRNA, Figure 4).

### 3.4 LRP8 depletion induces apoptosis

**Figure 5:**
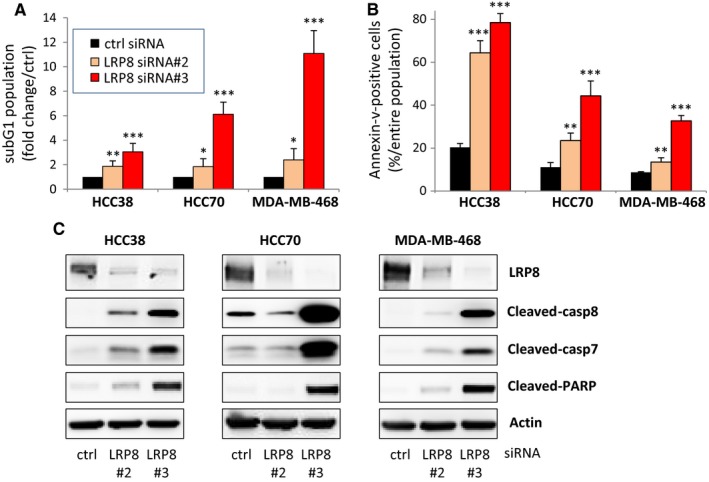
LRP8 depletion induces apoptosis. TNBC cells were transfected with one of the two siRNAs against LRP8 (#2 and #3: orange and red, respectively) or with a control siRNA (black). A, Percentage of sub‐G1 cells 144 h after siRNA treatment, in an analysis of cell cycle progression (Figure 4A). B, Annexin‐V‐positive cells 144 h after siRNA treatment. For the purposes of quantification (A, B), the data are expressed as the means ± SD from at least three independent experiments. *P* values determined with Student's *t* test (comparison with control siRNA) are indicated (**P < 0.01; ***P < 0.001). C, Western blot to evaluate caspase activity 144 h after LRP8 depletion. Antibodies recognizing the cleaved forms of caspase 7, caspase 8, and PARP were used. LRP8 depletion was verified with anti‐LRP8 antibodies. Actin was used as a loading control. The images shown are from a single experiment representative of three independent experiments performed

Our analyses of cell cycle progression indicated that the sub‐G1 population, potentially corresponding to apoptotic cells, was larger in all LRP8‐depleted cells than in control cells (Figures 4A and 5A). We performed additional experiments to determine whether LRP8 depletion did, indeed, induce apoptosis. We first measured phosphatidylserine levels on the surface of the cells, by annexin‐V staining of living cells. Phosphatidylserine exposure on the cell surface is one of the first events in apoptosis. In the three cell lines tested, LRP8 depletion resulted in larger numbers of annexin‐V‐positive cells than were observed for cells treated with control siRNA (Figure 5B). Annexin‐V staining was stronger in HCC38 cells than in the other cell lines after LRP8 depletion (Figure 5B). However, the fold change in the number of annexin‐V‐positive cells relative to the control siRNA condition was similar in all three cell lines. Again, the LRP8 siRNA#3 seemed to be the most effective, generating the largest number of apoptotic cells, consistent with its stronger effects on survival than the other LRP8 siRNAs. We also evaluated apoptosis by measuring caspase activity by Western blotting after LRP8 depletion (Figure 5C). An induction of the cleaved forms of caspase‐7, caspase‐8, and PARP was observed in cells from which LRP8 had been depleted (Figure 5C). Once again, caspase activity was more strongly induced by LRP8 siRNA#3 than by LRP8 siRNA#2, in all cell lines.

Overall, these results clearly demonstrate that LRP8 depletion leads to apoptosis, and, therefore, that this transmembrane receptor is essential for cell survival.

### 3.5 The depletion of LRP8 slows tumor growth in a xenograft model

**Figure 6:**
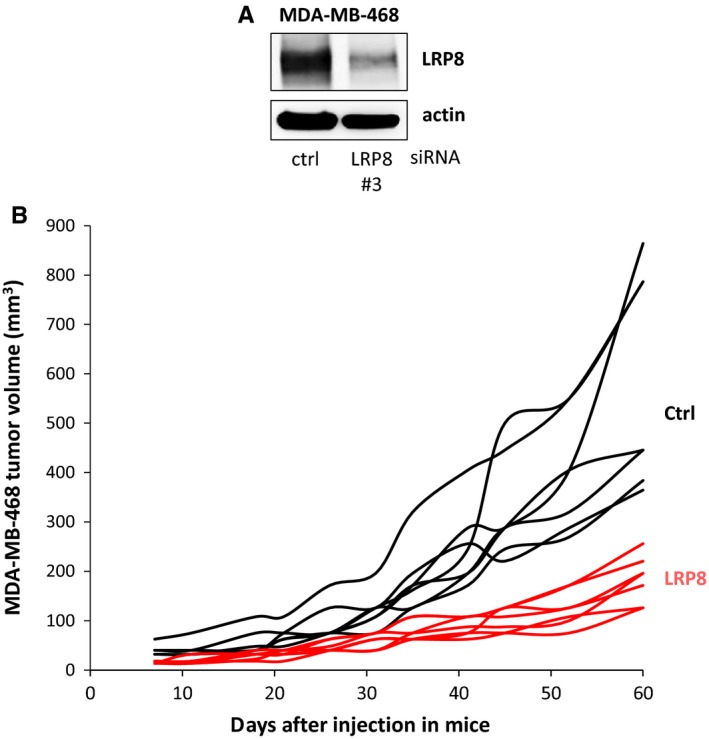
LRP8 depletion slows tumor growth. MDA‐MB‐468 cells were transfected with control (ctrl, black) or LRP8 (#3, red) siRNAs. A, The level of LRP8 protein was evaluated by Western blotting 24 h after transfection. Actin was used as a loading control. B, Twenty‐four hours after transfection, 4 × 10^6^ MDA‐MB‐468 cells were injected subcutaneously into Swiss *nude* mice (7 animals/group). Tumor growth was evaluated twice weekly for 2 mo. The data shown are the tumor volume measured for each animal. The differences in tumor volume between the control siRNA and LRP8 siRNA groups were evaluated at each time point, in Wilcoxon tests. We used Benjamini‐Hochberg correction to adjust for multiple testing. Differences were considered significant if the adjusted *P* value was below 0.05. This was the case for all time points from day 11 (adjusted *P* = 0.003)

As LRP8 was found to have tumorigenic properties in vitro*, *being required for anchorage‐independent growth (Figure 3B), we investigated the effect of LRP8 depletion on tumor growth in a xenograft model, by evaluating the tumorigenic potential of MDA‐MB‐468 cells following their injection into mice. Twenty‐four hours after the transfection of MDA‐MB‐468 cells with control or LRP8 siRNAs, we verified that the level of LRP8 had indeed been reduced by transfection with the LRP8 siRNA (Figure 6A), and we then injected equal numbers of cells into mice (Figure 6B). LRP8 depletion significantly decreased tumor growth (adjusted *P* = 0.003 at all time points after day 11), supporting that LRP8 is a potential candidate treatment target for TNBC.

## 4 DISCUSSION

LRP8 is a transmembrane receptor that has been extensively studied in the field of neuroscience, but its role in cancers is still largely unknown. A limited number of studies have begun to explore the role of the transmembrane receptor LRP8 in cancers: an amplification of the *LRP8* gene in squamous lung cancer,^18^ a role in migration though its expression in endothelial cells in melanoma,^19^ and more recently it has been shown that ApoE/LRP8 axis promotes antitumor immunity by targeting immunosuppressive innate immune cells.^20^ When writing the manuscript, we found that a study which was/is not referenced in PubMed showed that LRP8 expression was higher in ER^−^/HER2^−^ compared to ER^+^/HER2^−^ tumors, and that LRP8 depletion impaired the proliferation specifically of TNBC cell lines and not in breast cancer cell lines of other types.^21^ However, that study did not evaluate LRP8 expression in HER2‐positive breast cancer samples.

The objective of our study was to determine the levels of LRP8 in all major subtypes of breast cancers using patient samples and a large panel of breast cancer cell lines. We show for the first time that LRP8 RNA levels were higher in estrogen‐negative breast tumors (TNBC and HER2 [ER^−^/HER2^+^] tumors), than in luminal breast tumors (LA [ER^+^/HER2^−^] and LB [ER^+^/HER2^+^] samples), and that these levels were the highest in TNBC samples, in three different cohorts. Although a previous report showed that LRP8 expression was higher in ER^−^/HER2^−^ (our TNBC group) compared to ER^+^/HER2^−^ (our LA group) tumors,^21^ a complete analysis of all breast cancer subtypes allows us to consider LRP8 as a relevant therapeutic target for both ER^−^/HER2^+ ^and TNBC subtypes.

LRP8 expression in breast cancer cell lines was similar to that in biopsy specimens, with the highest levels observed in cell lines without ER expression, in contrast to the study of Arun et al^21^ reporting no differential expression by ER status. Transcriptomic analysis of an in‐house panel of 40 breast cancer cell lines also revealed no difference in RNA LRP8 expression between TNBC and luminal cell lines (data not shown). Therefore, the apparent discrepancy may come from the way of analyzing the expression of LRP8 in cell lines (RNA vs protein).

Some variability of LRP8 levels was observed between TNBC cell lines and between TNBC biopsy specimens. Given the considerable heterogeneity of TNBC,^3^, ^4^, ^5^, ^6^ we investigated whether LRP8 RNA levels were associated with a specific TNBC subtype. Using the updated Lehmann classification,^7^ we found that the LAR (luminal androgen receptor) TNBC subtype had lower levels of LRP8 RNA than the other TNBC subtypes, which had similar LRP8 levels to each other (data not shown). Accordingly, MDA‐MB‐453 cells, the only LAR cell line studied here, had low LRP8 protein levels. Overall, our results indicate that LRP8 is less strongly expressed in tumors that express hormone receptors (estrogen, progesterone, androgen receptors). The high levels of LRP8 in TNBC and ER^−^/HER2^+^ tumors suggested that these tumors might be dependent on LRP8 expression, and that it might be possible to eradicate these tumor cells by targeting LRP8. The identification of new treatments for TNBC patients is a matter of priority in oncology. We therefore focused on TNBC cells rather than ER^−^/HER2^+^ tumors, for which treatments targeting HER2 are already available.

By analyzing 13 breast cancer cell lines, we found that TNBC and ER^−^/HER2^+^ cell lines were more sensitive to LRP8 depletion than cell lines expressing ER However, analysis of additional ER^+^ cell lines should be performed in order to further substantiate this statement. We clearly demonstrate that LRP8 depletion affected the viability of all ER‐negative cells, not specifically TNBC cells as previously reported,^21^ but also ER^−^/HER2^+^ cells. We found that LRP8 depletion impaired cell viability by preventing cell cycle progression and inducing apoptosis in various TNBC cell lines, previously unreported. The molecular mechanism by which LRP8 controls cell survival is unknown, and further studies are required to resolve this issue. We also report for the first time that LRP8 depletion in TNBC cells renders these cells unable to form colonies in an anchorage‐dependent (growth on plastic) or anchorage‐independent (growth in soft agar) manner, the latter suggesting protumorigenic properties of LRP8. The potential tumorigenic properties of LRP8 were confirmed in vivo in a xenograft model, in which tumor growth was slowed by LRP8 depletion, confirming a previous study using another TNBC cell line (MDA‐MB‐231).^21^

Our collective findings suggest that LRP8 could be an attractive treatment target against TNBC. LRP8‐targeting strategies could include antibody approaches based on therapeutic monoclonal antibodies, for example, exploiting its role in tumor progression, or with antibody‐drug conjugates (ADC) to capitalize on its restricted pattern of expression. LRP8 was also identified in a computational study searching for transmembrane genes more strongly expressed in TNBCs than in various normal tissues for the development of an ADC approach.^35^ Antibodies targeting LRP8 (LRP8‐binding protein) have recently been patented by AbbVie, but their effects on cell survival or tumor growth have yet to be reported. Once anti‐LRP8 antibodies become available, evaluations in preclinical studies will be required to determine their effects on tumor growth in TNBC patient‐derived xenograft models, alone and in combination with the standard chemotherapies currently used to treat TNBC in clinical practice. Limited adverse effects could be expected as most normal tissues present low levels of LRP8, as reported.^21^ In normal tissues, the highest levels of LRP8 were detected in brain tissues, but an antibody‐based therapeutic approach would avoid potential neurotoxicity as antibodies do not easily cross the blood‐brain barrier. Given that cell lines expressing the estrogen or androgen receptors tended to be less sensitive to LRP8 knockdown, hormone receptor status may thus be a useful biomarker to stratify patients in prospective clinical trials targeting LRP8.

Overall, our results suggest LRP8 as a potential new treatment target in estrogen‐negative breast cancers, including those overexpressing HER2 and TNBCs, for which there currently is a clear and unmet clinical need.

## CONFLICT OF INTEREST

The authors have no conflicts of interest to declare.
