# Supplementary material for: LRP8 is overexpressed in estrogen‐negative breast cancers and a potential target for these tumors
Source: Cancer Med. 2018 Dec 21;8(1):325–36. doi: 10.1002/cam4.1923 (PMC6346259; doi:10.1002/cam4.1923)

Table S1. Number of cells seed per well for all cellular assays.

	proliferation assays	western-blot, apoptosis, cell cycle analysis	clonogenic assay	soft-agar assay
	**96 well-plates**	**6 well-plates**	**6 well-plates**	**6 well-plates**
**BT474**	12 000	400 000	-	-
**T47D**	5 000	100 000	-	-
**MCF7**	6 000	300 000	-	-
**ZR75.1**	6 000	300 000	-	-
**SKBR3**	6 000	300 000	-	-
**HCC1569**	5 000	150 000	-	-
**HCC1954**	5 000	150 000	-	-
**BT20**	6 000	200 000	-	-
**HCC1143**	5 000	150 000	-	-
**HCC38**	8 000	300 000	8 000	-
**HCC70**	10 000	400 000	10 000	5 000
**MDA-MB-468**	5 000	250 000	5 000	5 000
**MDA-MB-453**	8 000	300 000	-	-

Figure S1: GAPDH RNA expression in various normal tissues.

As in Figure 1C, GAPDH RNA levels were analyzed in a panel of normal tissues (black bars) and in five TNBC samples (red bars). GAPDH RNA levels are normalized relative to those in the adrenal gland (=1).

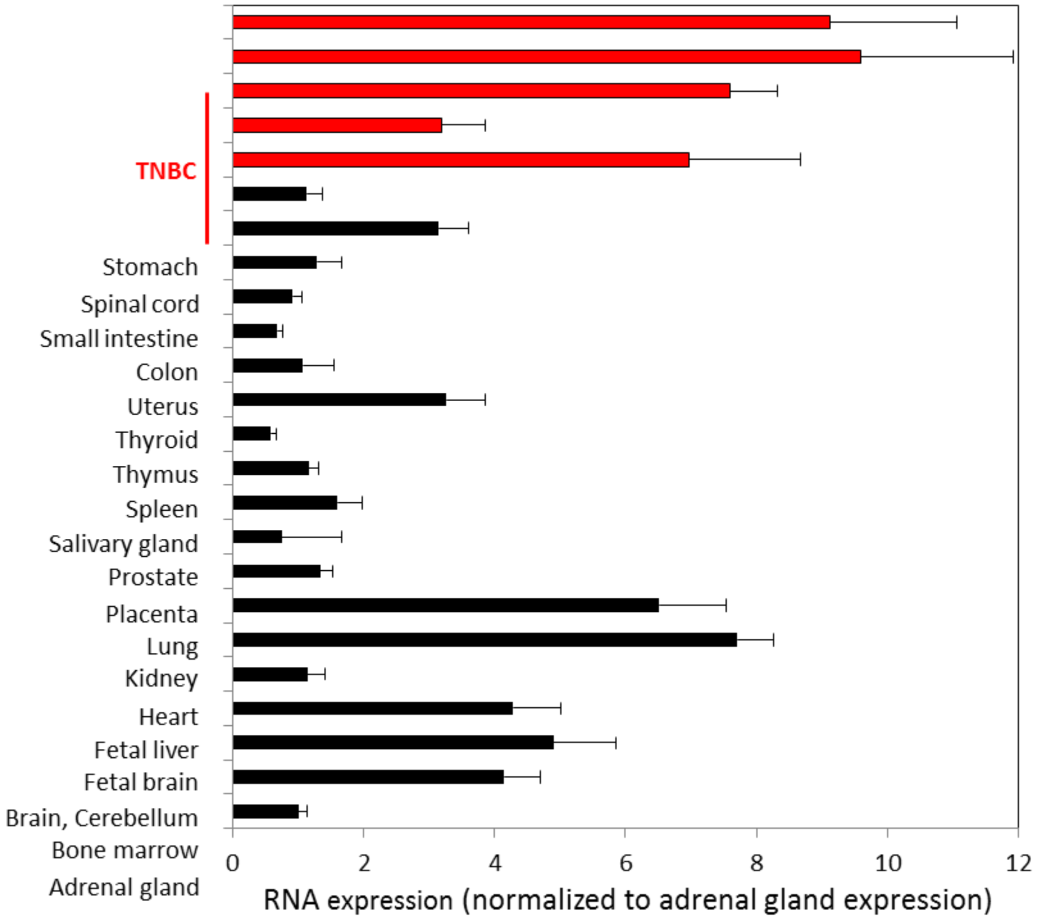

Figure S2: LRP8 is more strongly expressed in hormone receptor-negative breast tumors than in luminal cancers, and expression levels are highest in TNBC.

LRP8 RNA levels in the different breast cancer subtypes from the METABRIC cohort [ref.27 in the manuscript]. TNBC (red), HER2+/ER- (HER2, blue), luminal A (LA, orange), and luminal B (LB, green) cancers. The relative levels of RNA have been subjected to a logarithmic (log2) transformation and are illustrated by boxplots. Outliers are shown within each population studied (open circles). Student's t test was used to compare RNA levels between two groups. The P values are indicated (***P* < 0.01; ***P < 0.001).

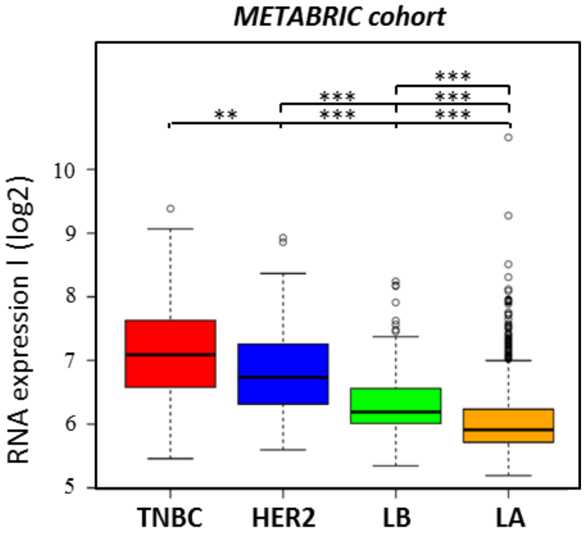

Supplement: Supplementary file 1 [file CAM4-8-325-s001.pdf]
